# Supplementary material for: Penetrance of Hemochromatosis in HFE Genotypes Resulting in p.Cys282Tyr and p.[Cys282Tyr];[His63Asp] in the eMERGE Network
Source: Am J Hum Genet. 2015 Sep 10;97(4):512–20. doi: 10.1016/j.ajhg.2015.08.008 (PMC4596892; doi:10.1016/j.ajhg.2015.08.008)
Supplement: Document S1. Tables S1–S4 [file mmc1.pdf]

The American Journal of Human Genetics
Supplemental Data

Penetrance of Hemochromatosis in *HFE* Genotypes Resulting in p.Cys282Tyr and p.[Cys282Tyr];[His63Asp] in the eMERGE Network

Carlos J. Gallego, Amber Burt, Agnes S. Sundaresan, Zi Ye, Christopher Shaw, David R. Crosslin, Paul K. Crane, S. Malia Fullerton, Kris Hansen, David Carrell, Helena Kuivaniemi, Kimberly Derr, Mariza de Andrade, Catherine A. McCarty, Terrie E. Kitchner, Brittany K. Ragon, Sarah C. Stallings, Gabriella Papa, Joseph Bochenek, Maureen E. Smith, Sharon A. Aufox, Jennifer A. Pacheco, Vaibhav Patel, Elisha M. Friesema, Angelika Ludtke Erwin, Omri Gottesman, Glenn S. Gerhard, Marylyn Ritchie, Arno G. Motulsky, Iftikhar J. Kullo, Eric B. Larson, Gerard Tromp, Murray H. Brilliant, Erwin Bottinger, Joshua C. Denny, Dan M. Roden, Marc S. Williams, and Gail P. Jarvik

Table S1. Genotyped versus imputed variants by site. Number of samples that were genotyped or imputed for *HFE* variants p.Cys282Tyr and p.His63Asp by site. [a] Only p.Cys282Tyr was genotyped, p.His63Asp was imputed. ; [b] Only p.His63Asp was genotyped, p.Cys282Tyr was imputed.; [c] Both p.Cys282Tyr and p.His63Asp were imputed.

		GENOTYPED SNP			
SITE	BOTH	p.Cys282Tyr[a]	p.His63Asp[b]	NEITHER[c]	TOTAL
Geisinger	974	0	3111	0	4085
Group Health/UW	0	3130	398	0	3528
Marshfield	0	4193	0	0	4193
Mayo	14	6862	0	0	6876
Mt. Sinai	0	2775	3515	0	6290
Northwestern	605	1302	0	2951	4858
Vanderbilt	2082	4004	0	3461	9547
Total	3675	22266	7024	6412	39377

Table S2. Examples of data obtained in chart abstraction, by category. The list is in the order of information usually present in medical records.

DATA CATEGORY	EXAMPLES
Demographic	Age at last observation, vital status, sex, race, ethnicity
Medical history	Hemochromatosis, iron-overload, liver cirrhosis, chronic liver diseases, secondary causes of hemochromatosis, congestive heart failure, cardiomyopathy, diabetes, arthritis, hypogonadism
Laboratories	Transferrin saturation, serum ferritin, serum iron, total iron binding capacity, liver transaminases, prothrombin time, partial thromboplastin time, follicle-stimulating hormone, luteinizing hormone, testosterone
Imaging	Hand x-ray, echocardiogram, cardiac magnetic resonance imaging, abdominal ultrasound, abdominal computed tomography, electrocardiogram
Family history	Relatives affected with hemochromatosis, number and degree of relationship
Past social history	Alcoholism, tobacco abuse, intravenous drug use
Medications	Tylenol, other arthritis medications, iron supplements, diabetes medications
Physical exam	Hepatomegaly, ascites, signs of congestive heart failure, arthritis, skin pigmentation (bronze skin)

Table S3. List of covariates in the analysis, stratified by genotype and sex. The list is in the order of information usually present in medical records: medical, family, social history, medications, review of systems, imaging studies, laboratories, and physical findings. Italicized text corresponds to liver biopsy characteristics. Unless otherwise specified, data are represented as percentage, with the proportion of affected patients in parentheses. Abbreviations: NA, not available; AST, aspartate aminotransferase; ALT, alanine aminotransferase; HH, hereditary hemochromatosis; PIP/MCP, proximal interphalangeal/metacarpophalangeal. Footnotes: [a] Diagnosed by notes, ICD-9 code 275.0 or 275.09 or lab criteria: transferrin saturation (> 45%) and serum ferritin (>200 ng/mL in men and >150 ng/mL in women), [b] Diagnosed by notes, ICD-9 code 571, [c] Diagnosed by notes, ICD-9 codes 571, 794.8, 790.4, 790.6, lab criteria: liver enzyme elevation for >6 months, [d] Diagnosed by notes, ICD-9 codes 280.9 or 275.0, lab criteria: ferritin <40 ng/mL and anemia or ICD-9 codes 281 or 285.

	p.Cys282Tyr/p.Cys282Tyr (N=98)			p.Cys282Tyr/p.His63Asp (N=397)		
VARIABLE	N	MALE (N=47)	FEMALE (N=51)	N	MALE (N=175)	FEMALE (N=222)
Body Mass Index	83	29.0±5.4	32.3±23.5	359	29.0±4.9	29.4±8.2
Liver Biopsy (any liver biopsy)	90	10.9% (5/46)	9.1% (4/44)	371	1.8% (3/166)	16.1% (33/205)
Liver Biopsy (not incidental to gastric bypass)	90	10.9% (5/46)	9.1% (4/44)	371	1.8% (3/166)	2.0% (4/205)
Cirrhosis in Liver Biopsy Results	6	0.0% (0/3)	33.3% (1/3)	38	0.0% (0/4)	5.9% (2/34)
Normal Liver Biopsy Results	5	0% (0/3)	50% (1/2)	35	0.0% (0/3)	18.8% (6/32)
Iron Deposition in Liver Biopsy Results	7	33.3% (1/3)	50.0% (2/4)	35	66.7% (2/3)	28.1% (9/32)
Iron Overload [a]	88	30.4% (14/46)	9.5% (4/42)	375	3.5% (6/170)	2.9% (6/205)

Liver Cirrhosis [b]	84	4.5% (2/44)	2.5% (1/40)	369	4.8% (8/166)	4.9% (10/203)
Other Chronic Liver Disease Causes [c]	84	7% (3/43)	0% (0/41)	369	6.7% (11/164)	7.8% (16/205)
Iron Deficiency [d]	86	15.6% (7/45)	4.9% (2/41)	371	7.1% (12/168)	10.8% (22/203)
Phlebotomy	96	19.6% (9/46)	8.0% (4/50)	391	2.9% (5/172)	0.5% (1/219)
Congestive Heart Failure	95	21.7% (10/46)	18.4% (9/49)	392	16.8% (29/173)	8.7% (19/219)
Cardiomyopathy	94	6.7% (3/45)	4.1% (2/49)	392	7.5% (13/174)	1.8% (4/218)
Coronary Artery Disease	92	29.5% (13/44)	12.5% (6/48)	388	28.1% (48/171)	11.1% (24/217)
Diabetes	97	44.7% (21/47)	12.0% (6/50)	395	28.0% (49/175)	19.5% (43/220)
Arthritis	94	29.5% (13/44)	26.0% (13/50)	391	35.3% (61/173)	30.3% (66/218)
Hepatocellular Carcinoma	96	0% (0/46)	0% (0/50)	387	0% (0/169)	0% (0/218)
Family History of HH	82	8.1% (3/37)	6.7% (3/45)	356	0.0% (0/157)	1.5% (3/199)
Alcohol Dependence	94	13.3% (6/45)	2.0% (1/49)	381	10.6% (18/170)	7.1% (15/211)
Tobacco Use	94	30.4% (14/46)	16.7% (8/48)	391	31.6% (55/174)	23.0% (50/217)
Intravenous Drug Use	84	0% (0/40)	0% (0/44)	346	0.7% (1/150)	1.0% (2/196)
Over the counter Arthritis Medication	97	17% (8/47)	24% (12/50)	385	20.0% (34/170)	24.2% (52/215)
Acetaminophen	97	26.1% (12/46)	45.1% (23/51)	388	32.2% (55/171)	39.2% (85/217)
Prescription Arthritis Medication	95	25.0% (11/44)	17.6% (9/51)	384	30.4% (51/168)	26.9% (58/216)
Erectile Dysfunction Medication	47	19.1% (9/47)	NA	172	21.5% (37/172)	NA
Insulin	98	17.0% (8/47)	3.9% (2/51)	394	14.9% (26/174)	13.6% (30/220)
Oral Diabetes Therapy	98	27.7% (13/47)	7.8% (4/51)	391	18.6% (32/172)	16.0% (35/219)
Chronic Weakness/Lethargy	89	14.9% (7/47)	16.7% (7/42)	371	5.3% (9/169)	9.9% (20/202)
Arthralgia	90	25.5% (12/47)	23.3% (10/43)	373	24.0% (41/171)	21.8% (44/202)
Impotence	45	26.7% (12/45)	NA	158	16.5% (26/158)	NA
Abdominal Pain	87	17.8% (8/45)	7.1% (3/42)	365	9.6% (16/166)	18.1% (36/199)
Hepatomegaly	87	2.2% (1/46)	0.0% (0/41)	366	3.6% (6/165)	2.5% (5/201)
Ascites	88	4.3% (2/46)	0.0% (0/42)	368	1.8% (3/167)	2.5% (5/201)
Pain on palpation PIP/MCP joints	88	6.5% (3/46)	7.1% (3/42)	367	3% (5/166)	3% (6/201)

Skin Pigmentation	87	4.4% (2/45)	2.4% (1/42)	368	1.2% (2/167)	2.5% (5/201)
Hypogonadism	45	2.2% (1/45)	NA	167	1.8% (3/167)	NA
Erectile Dysfunction	42	23.8% (10/42)	NA	166	27.1% (45/166)	NA
Testicular Atrophy	45	2.2% (1/45)	NA	161	0% (0/161)	NA
Gynecomastia	43	0% (0/43)	NA	161	1.2% (2/161)	NA
Hand X-Ray	95	10.9% (5/46)	24.5% (12/49)	390	18.5% (32/173)	11.5% (25/217)
Echocardiogram	95	52.2% (24/46)	44.9% (22/49)	387	58.5% (100/171)	42.1% (91/216)
Cardiac Magnetic Resonance Imaging	96	2.2% (1/46)	0.0% (0/50)	390	0.6% (1/173)	0.9% (2/217)
Abdominal Ultrasound	94	25% (11/44)	26% (13/50)	392	24.7% (43/174)	34.4% (75/218)
Cirrhosis on Abdominal Ultrasound	25	0% (0/11)	0% (0/14)	118	4.7% (2/43)	2.7% (2/75)
Computed Tomography of Abdomen	96	41.3% (19/46)	26.0% (13/50)	389	46.5% (80/172)	37.3% (81/217)
Electrocardiogram	94	79.1% (34/43)	78.4% (40/51)	384	90.5% (153/169)	83.3% (179/215)
Highest AST (u/L)	87	41.5±20.1	50.7±82.6	344	87.4±354.1	51.3±94.1
AST > 80 u/L	87	2.5% (1/40)	8.5% (4/47)	344	12.4% (19/153)	8.9% (17/191)
Highest ALT (u/L)	74	43.3±23.7	68.0±164.2	291	66.7±197.4	57.8±115.6
ALT > 110 u/L	74	0.0% (0/35)	5.1% (2/39)	291	7.5% (10/133)	8.2% (13/158)
Rales on lung exam	87	13.3% (6/45)	14.3% (6/42)	371	6.0% (10/168)	6.4% (13/203)
Lower Extremity Edema	88	17.4% (8/46)	19.0% (8/42)	370	18.5% (31/168)	15.3% (31/202)
Transferrin Sat > 45%	17	100.0% (9/9)	62.5% (5/8)	56	37.5% (6/16)	40.0% (16/40)
Transferrin Sat > 50%	17	100% (9/9)	50% (4/8)	56	37.5% (6/16)	37.5% (15/40)
Highest Transferrin Saturation (%)	17	92.8±4.5	54.9±30.3	56	53.7±52.0	43.2±23.8
Highest Serum Iron Concentration μg/dL	33	186.8±57.8	144.6±58.3	104	127.5±63.2	127.8±60.5
Highest Total Iron-Binding Capacity (ng/mL)	33	269.1±55.5	269.4±42.9	93	288.4±72.8	317.3±61.8
Ferritin > 150 ng/mL	31	88.9% (16/18)	46.2% (6/13)	77	50.0% (12/24)	41.5% (22/53)
Ferritin > 200 ng/mL	31	88.9% (16/18)	30.8% (4/13)	77	45.8% (11/24)	30.2% (16/53)

Ferritin > 300 ng/mL	31	77.8% (14/18)	23.1% (3/13)	77	33.3% (8/24)	18.9% (10/53)
Ferritin > 1000 ng/mL	31	27.8% (5/18)	0.0% (0/13)	77	8.3% (2/24)	1.9% (1/53)
Highest Ferritin Level (ng/mL)	31	762.4±652.6	225.3±218.6	77	316.6±394.0	451.3±1891.9
Highest INR	59	2.8±2.3	2.0±1.2	193	2.2±1.7	2.2±1.8

Table S4. Penetrance of hereditary hemochromatosis by *HFE* genotype and eMERGE site. Data are represented as percentage, with the proportion of affected patients in parentheses.

	MALE		FEMALE	
SITE	p.Cys282Tyr/ p.Cys282Tyr	p.Cys282Tyr/ p.His63Asp	p.Cys282Tyr/ p.Cys282Tyr	p.Cys282Tyr/ p.His63Asp
Geisinger	0% (0/9)	4.4% (1/23)	0% (0/2)	2.6% (1/38)
Group Health/UW	33.3% (1/3)	0% (0/21)	12.5% (1/8)	0% (0/25)
Marshfield	42.9% (3/7)	3.6% (1/28)	25.0% (2/8)	0% (0/23)
Mayo	35.7% (5/14)	3.3% (2/61)	20.0% (3/15)	1.9% (1/53)
Mt Sinai	0% (0/0)	0% (0/0)	0% (0/0)	0% (0/0)
Northwestern	33.3% (1/3)	14.3% (1/7)	14.3% (1/7)	2.6% (1/39)
Vanderbilt	11.1% (1/9)	2.9% (1/34)	0% (0/10)	5.0% (2/40)
Total	24.4% (11/45)	3.5% (6/174)	14.0% (7/50)	2.3% (5/218)
