# Supplementary material for: Penetrance of Hemochromatosis in HFE Genotypes Resulting in p.Cys282Tyr and p.[Cys282Tyr];[His63Asp] in the eMERGE Network
Source: Am J Hum Genet. 2015 Sep 10;97(4):512–20. doi: 10.1016/j.ajhg.2015.08.008 (PMC4596892; doi:10.1016/j.ajhg.2015.08.008)
Supplement: Document S2. Article plus Supplemental Data [file mmc2.pdf]

Penetrance of Hemochromatosis in *HFE* Genotypes Resulting in p.Cys282Tyr and p.[Cys282Tyr];[His63Asp] in the eMERGE Network

Carlos J. Gallego,[1,2,23,*] Amber Burt,[1,23] Agnes S. Sundaresan,[3] Zi Ye,[4] Christopher Shaw,[1] David R. Crosslin,[1] Paul K. Crane,[5] S. Malia Fullerton,[6] Kris Hansen,[7] David Carrell,[7] Helena Kuivaniemi,[8] Kimberly Derr,[8] Mariza de Andrade,[9] Catherine A. McCarty,[10] Terrie E. Kitchner,[11] Brittany K. Ragon,[12] Sarah C. Stallings,[13] Gabriella Papa,[13] Joseph Bochenek,[14] Maureen E. Smith,[15] Sharon A. Aufox,[15] Jennifer A. Pacheco,[15] Vaibhav Patel,[15] Elisha M. Friesema,[16] Angelika Ludtke Erwin,[17] Omri Gottesman,[17] Glenn S. Gerhard,[18] Marylyn Ritchie,[19] Arno G. Motulsky,[1] Iftikhar J. Kullo,[4] Eric B. Larson,[7] Gerard Tromp,[8] Murray H. Brilliant,[11] Erwin Bottinger,[17] Joshua C. Denny,[14,20] Dan M. Roden,[20,21] Marc S. Williams,[22] and Gail P. Jarvik[1,24,*]

Hereditary hemochromatosis (HH) is a common autosomal-recessive disorder associated with pathogenic *HFE* variants, most commonly those resulting in p.Cys282Tyr and p.His63Asp. Recommendations on returning incidental findings of *HFE* variants in individuals undergoing genome-scale sequencing should be informed by penetrance estimates of HH in unselected samples. We used the eMERGE Network, a multicenter cohort with genotype data linked to electronic medical records, to estimate the diagnostic rate and clinical penetrance of HH in 98 individuals homozygous for the variant coding for HFE p.Cys282Tyr and 397 compound heterozygotes with variants resulting in p.[His63Asp];[Cys282Tyr]. The diagnostic rate of HH in males was 24.4% for p.Cys282Tyr homozygotes and 3.5% for compound heterozygotes ($p < 0.001$); in females, it was 14.0% for p.Cys282Tyr homozygotes and 2.3% for compound heterozygotes ($p < 0.001$). Only males showed differences across genotypes in transferrin saturation levels (100% of homozygotes versus 37.5% of compound heterozygotes with transferrin saturation > 50%; $p = 0.003$), serum ferritin levels (77.8% versus 33.3% with serum ferritin > 300 ng/ml; $p = 0.006$), and diabetes (44.7% versus 28.0%; $p = 0.03$). No differences were found in the prevalence of heart disease, arthritis, or liver disease, except for the rate of liver biopsy (10.9% versus 1.8% [$p = 0.013$] in males; 9.1% versus 2% [$p = 0.035$] in females). Given the higher rate of HH diagnosis than in prior studies, the high penetrance of iron overload, and the frequency of at-risk genotypes, in addition to other suggested actionable adult-onset genetic conditions, opportunistic screening should be considered for p.[Cys282Tyr];[Cys282Tyr] individuals with existing genomic data.

Introduction

Hereditary hemochromatosis (HH [MIM: 235200]) is the most common genetic disorder identified in those of European ancestry and is characterized by an inappropriate increased absorption of dietary iron. If left untreated, HH can lead to morbidity and mortality, including liver cirrhosis, hepatocellular carcinoma, diabetes, and heart disease.[1] If treatment with regular phlebotomy is initiated before organ damage develops, these complications can be prevented, and people with HH can have a normal life expectancy.[2] Variants in *HFE* (MIM: 613609; GenBank: NM_000410.3) are associated with the majority of adult-onset HH cases; the pathogenic variant with the highest penetrance is c.845G>A, a missense mutation (minor allele frequency [MAF] = 4% in the 1000 Genomes European population) that results in the substitution of tyrosine for cysteine in the protein product (p.Cys282Tyr) and accounts

[1]Division of Medical Genetics, Department of Medicine, University of Washington, Seattle, WA 98195, USA; [2]Pharmaceutical Outcomes Research and Policy Program, Department of Pharmacy, University of Washington, Seattle, WA 98195, USA; [3]Center for Health Research, Geisinger Health System, Danville, PA 17822, USA; [4]Division of Cardiovascular Diseases, Department of Internal Medicine, Mayo Clinic, Rochester, MN 55905, USA; [5]Division of General Internal Medicine, Department of Medicine, University of Washington, Seattle, WA 98104, USA; [6]Department of Bioethics and Humanities, University of Washington, Seattle, WA 98195, USA; [7]Group Health Research Institute, Group Health Cooperative, Seattle, WA 98101, USA; [8]Siegfried and Janet Weis Center for Research, Geisinger Health System, Danville, PA 17822, USA; [9]Division of Biomedical Statistics and Informatics, Department of Health Sciences Research, Mayo Clinic, Rochester, MN 55905, USA; [10]Research Division, Essentia Institute of Rural Health, Duluth, MN 55805, USA; [11]Center for Human Genetics, Marshfield Clinic Research Foundation, Marshfield, WI 54449, USA; [12]Division of Cancer Medicine, MD Anderson Cancer Center, Houston, TX 77030, USA; [13]Vanderbilt Institute for Clinical and Translational Research, Vanderbilt University School of Medicine, Nashville, TN 37203, USA; [14]Department of Biomedical Informatics, Vanderbilt University School of Medicine, Nashville, TN 37232, USA; [15]Center for Genetic Medicine, Feinberg School of Medicine, Northwestern University, Chicago, IL 60611, USA; [16]Division of General Internal Medicine and Geriatrics, Feinberg School of Medicine, Northwestern University, Chicago, IL 60611, USA; [17]Charles Bronfman Institute for Personalized Medicine, Icahn School of Medicine at Mount Sinai, New York, NY 10029, USA; [18]Department of Medical Genetics and Molecular Biochemistry, Temple University School of Medicine, Philadelphia, PA 19140, USA; [19]Department of Biochemistry and Molecular Biology, Pennsylvania State University, University Park, PA 16802, USA; [20]Department of Medicine, Vanderbilt University School of Medicine, Nashville, TN 37232, USA; [21]Department of Pharmacology, Vanderbilt University School of Medicine, Nashville, TN 37232, USA; [22]Genomic Medicine Institute, Geisinger Health System, Danville, PA 17822, USA
[23]These authors contributed equally to this work
[24]Present address: 1705 Northeast Pacific Street, K253, Box 357720, Seattle, WA 98195-7720, USA
*Correspondence: cgallego@med.umich.edu (C.J.G.), gjarvik@medicine.washington.edu (G.P.J.)
http://dx.doi.org/10.1016/j.ajhg.2015.08.008. ©2015 The Authors
This is an open access article under the CC BY license (http://creativecommons.org/licenses/by/4.0/).

for 80%–85% of individuals with HH.[3,4] A more common HH variant (MAF = 17% in the 1000 Genomes European population) is c.187C>G, which results in the substitution of aspartate for histidine at amino acid position 63 (p.His63Asp); this variant causes a milder degree of iron overabsorption and is most relevant to disease when paired with the allele for p.Cys282Tyr in compound heterozygotes.[3] To improve clarity, we will henceforth use the protein change as a surrogate for genotype. Several other hemochromatosis-associated pathogenic variants in *HFE* have been described, as well as variants in genes other than *HFE* (e.g., *HJV* [MIM: 608374], *HAMP* [MIM: 606464], and *TFR2* [MIM: 604720]), but these are rare.[5,6]

Estimates of penetrance for HH-related variants vary widely depending on the signs or symptoms used in disease assessment.[7] For instance, in p.Cys282Tyr homozygotes from a racially diverse cohort, the prevalence of elevated serum ferritin (>300 ng/ml for males and >200 ng/ml for females) and transferrin saturation levels (>50% in men and >45% in women) was 40%–60% in females and 75%–100% in males.[8–12] In contrast, in another cohort of northern European descent, the prevalence of iron-overload-related disease (defined as liver fibrosis, elevated transaminases, hepatocellular carcinoma, arthropathy, or physician-diagnosed HH in individuals with high ferritin or transferrin saturation) in p.Cys282Tyr homozygotes was 1.2% (95% confidence interval [CI] = 0.03–6.5) in females and 28.4% (95% CI = 18.8–40.2) in males.[13] The lower clinical penetrance in women has been suggested to result from iron loss through menstrual bleeding and childbirth, although evidence is lacking.[5]

These variations in estimates of penetrance have resulted in insufficient evidence for confidently projecting the impact, or estimating the benefit, of widespread genetic screening for HH.[14] Whereas newborn screening for HH has not been adopted because of its adult onset and incomplete penetrance,[15] the utility of using existing genetic data for screening for HH risk has not been carefully addressed. The American College of Medical Genetics and Genomics (ACMG) does not currently include HH among the gene-disease pairs it recommends evaluating and returning as incidental findings for genome-scale sequencing,[16] although other recommendations have been proposed.[17] Development of such recommendations would be informed by the penetrance and rate of diagnosis of HH in unselected samples.

The primary objective of this study was to determine the frequency of diagnosis of *HFE*-related HH and to estimate the penetrance of clinically related variables in the Electronic Medical Records and Genomics (eMERGE) Network, a national consortium organized by the National Human Genome Research Institute to develop, disseminate, and apply approaches to research by combining DNA biorepositories with electronic-medical-record systems for high-throughput genetic research. The network's goals include returning genomic testing results to individuals in a clinical setting.[18,19] This cohort was generally ascertained independently of HH diagnosis. By identifying all participants carrying the p.[Cys282Tyr];[Cys282Tyr] and p.[Cys282Tyr];[His63Asp] genotypes and reviewing their medical records, we obtained a minimally biased estimate of general population frequency of HH diagnosis and related signs in those at genetic risk. A secondary objective of this study was to serve as a proof of principle to determine the efficacy of this multicenter consortium to estimate the penetrance of common phenotypes associated with relatively uncommon genetic variants.

Subjects and Methods

eMERGE Network Participants

The eMERGE Network is a consortium of seven adult and two pediatric US cohorts with DNA biorepositories linked to electronic-medical-record data for large-scale, high-throughput genetic research.[18–20] Participating sites for the adult cohort included the following: (1) Group Health Cooperative and University of Washington, Seattle, WA; (2) Marshfield Clinic, Marshfield, WI; (3) Mayo Clinic, Rochester, MN; (4) Northwestern University, Evanston, IL; (5) Vanderbilt University, Nashville, TN; (6) Icahn School of Medicine at Mount Sinai, New York, NY; and (7) Geisinger Health System, Danville, PA.[20] Northwestern's cohort included participants ascertained from a liver clinic; these participants were excluded from the calculation of HH penetrance. The Geisinger cohort included participants from a weight-loss and gastric-bypass clinic, which included liver biopsy in its routine care. These individuals were excluded from the calculation of the rate of liver biopsy. Because MAFs for rs1800562 and rs1799945 are more common in individuals of European ancestry and HH is most common in this ancestry, we limited our analysis to this population. The human-subjects procedures that we followed were in accordance with the ethical standards of the responsible committee on human experimentation at each institution, and proper informed consent was obtained from each participant.

Genotyping and Imputation

We selected participants who were either homozygotes for rs1800562 (p.Cys282Tyr) or compound heterozygotes for both rs1800562 and rs1799945 (p.His63Asp). Genotypes for these *HFE* variants were either directly measured or imputed for all sites.

Genotyping, quality-control, and imputation procedures for the eMERGE Network have been previously described.[21,22] Participants from eMERGE phase I were genotyped on the Illumina Human 660W-Quadv1_A or Illumina Human 1 M-Duo platform. Genotypes for both platforms were called at the Center for Inherited Disease Research and the Broad Institute with BeadStudio version 3.3.7 and Gentrain version 1.0. eMERGE phase II includes genotype data from a variety of platforms: Illumina 550, 610Q, 660W Quad-v1, 1M-Duo, 1M-Quad, OmniExpress, Metabochip, OMNI-1, OMNI-5, and Affymetrix 6.0.[19]

We performed imputation with a reference panel from the October 2011 release of the 1000 Genomes Project by using BEAGLE version 3.3.1. Both SNPs were genotyped on the Illumina Metabochip and 1M-Duo, rs1800562 was genotyped on the Illumina 660W, and rs1799945 was genotyped on the Illumina OMNI. For all other genotyping platforms, the imputed genotypes for the SNPs were used. Directly genotyped SNPs and imputed SNPs are summarized by site in Table S1.

Table 1. Site Distribution and Demographic Characteristics of Individuals by Genotype

	p.[Cys282Tyr];[Cys282Tyr]	p.[Cys282Tyr];[His63Asp]	Total
**No. of Samples per Site**			
Geisinger Health System	12	61	73
Group Health Cooperative and UW	12	48	60
Marshfield Clinic	15	52	67
Mayo Clinic	30	117	147
Icahn School of Medicine at Mount Sinai	1	13	14
Northwestern University	16	61	77
Vanderbilt University	20	80	100
Total	106	432	538
**Sample Descriptions**			
Proportion of males	45.28% (48/106)	44.91% (194/432)	44.98% (242/538)
Age at time of abstraction	67.5 ± 17.1	66.1 ± 15.5	66.4 ± 15.8
Age at diagnosis of HH	61.5 ± 14.6	56.3 ± 7.2	59.6 ± 12.5
**Race**			
White	92.45% (98/106)	91.90% (397/432)	92.00% (495/538)
African American	0% (0/106)	0.46% (2/432)	0.37% (2/538)
Other, unknown, or missing	7.55% (8/106)	7.64% (33/432)	7.62% (41/538)

For individuals with the *HFE* genotypes resulting in p.[Cys282Tyr];[Cys282Tyr] or p.[Cys282Tyr];[His63Asp] and available linked data from electronic medical records. Sex (males) and race data are represented as a percentage, and the proportion of affected individuals is in parentheses. Age is represented as the mean in years ± SD. Only the 495 individuals of European ancestry were included in the analysis. The following abbreviation is used: UW, University of Washington.

We genotyped the two *HFE* SNPs with the TaqMan at Geisinger Health System in a subset of 179 eMERGE participants to evaluate the concordance between the imputed genotypes and TaqMan genotypes. The concordance between TaqMan and imputed genotypes was 98.9% for rs1800562 and 98.3% for rs1799945.

Clinical Data

A chart-abstraction form was designed in accordance with published recommendations.[23] This common collection instrument was developed with a Research Electronic Data Capture form shared between all sites.[24] Electronic and paper medical records were reviewed. Demographic data were extracted from existing eMERGE databases. The information extracted from medical records included medical history of hemochromatosis or iron-overload-related conditions, iron-related laboratory studies, imaging studies, and physical findings consistent with HH (Table S2).

In the absence of secondary iron-overload state, HH diagnosis was based on the physician annotation of HH in the medical records, including a recent clinical note, problem list, or ICD-9 (International Code of Diseases, Ninth Revision) codes 275.01, 275.02, or 275.03. We used a broad definition of liver disease and included individuals with liver cirrhosis, other chronic hepatic phenotypes (i.e., alcoholic liver disease, chronic viral hepatitis, fatty liver disease, or non-specific liver enzyme elevation), hepatocellular carcinoma, hepatomegaly, ascites, history of liver biopsy, and elevated liver function. A manual of procedures was written for the data abstractors' training and reference, and common examples of situations encountered by abstractors were discussed before the final form was distributed. A pilot test form was designed to ensure the reliability and validity of the data-collection instruments. Monthly teleconferences with all sites took place during the form-development and abstraction phases of the project. These calls addressed questions about coding to ensure consistency, accounting for differences in medical-record structures (including different measuring units in the laboratories), and disparities in the availability of certain data.

Analysis

All statistical analyses and penetrance calculations were done in R statistical computing software. We stratified by sex and compared p.Cys282Tyr homozygous to p.[Cys282Tyr];[His63Asp] compound-heterozygous genotypes. Differences in the rates of HH diagnosis between genotypes were tested with a Chi-square test. Other HH-related phenotypes and related clinical and laboratory values between genotypes were tested with Fisher's exact test because of small sample sizes for some of the variables. Because the tests are correlated rather than independent, they were not adjusted for multiple comparisons.

Results

In the cohort of approximately 39,000 individuals with genotype data available in the eMERGE Network, we identified 618 individuals with the p.Cys282Tyr homozygous or p.[Cys282Tyr];[His63Asp] compound-heterozygous genotype; of these, 538 individuals had corresponding data from electronic medical records. Demographic characteristics of these individuals are summarized in Table 1. The mean age of participants was 66.4 years (±15.8), and the average age at HH diagnosis was 59.6 years (±12.5). The subsequent analysis included a total of 495 individuals of European ancestry (92%), 98 with the p.[Cys282Tyr];[Cys282Tyr] and 397 with the p.[Cys282Tyr];[His63Asp] genotype. We excluded six Northwestern participants who were ascertained from a liver clinic from the calculation of HH penetrance; 95 p.Cys282Tyr homozygotes and 392 compound heterozygotes with information on HH diagnosis were included in this analysis.

The frequency of HH diagnosis was 24.4% in male p.Cys282Tyr homozygotes and 3.5% in male p.[Cys282Tyr];[His63Asp] compound heterozygotes ($p < 0.001$), whereas the diagnostic rate was 14.0% in female p.Cys282Tyr homozygotes and 2.3% in female compound heterozygotes ($p < 0.001$). A summary of the differences in diagnostic rate and relevant phenotypes of HH across genotypes is

Table 2. Summary of Clinical Penetrance and Diagnostic Rate of HH

	Male (n = 222)			Female (n = 273)		
	p.[Cys282Tyr];[Cys282Tyr] (n = 47)	p.[Cys282Tyr];[His63Asp] (n = 175)	p Value	p.[Cys282Tyr];[Cys282Tyr] (n = 51)	p.[Cys282Tyr];[His63Asp] (n = 222)	p Value
**Clinical**						
Diagnostic rate of HH	24.4% (11/45)	3.4% (6/174)	<0.001	14.0% (7/50)	2.3% (5/218)	<0.001
Liver disease	34.3% (12/35)	24.4% (29/119)	0.279	29.0% (9/31)	29.0% (42/145)	1
Liver biopsy (not incidental to gastric bypass)	10.9% (5/46)	1.8% (3/166)	0.013	9.1% (4/44)	2.0% (4/205)	0.035
Liver cirrhosis[a]	4.5% (2/44)	4.8% (8/166)	1	2.5% (1/40)	4.9% (10/203)	0.697
Other chronic liver disease[b]	7.0% (3/43)	6.7% (11/164)	1	0% (0/41)	7.8% (16/205)	0.081
Hepatocellular carcinoma	0% (0/46)	0% (0/169)	NA	0% (0/50)	0% (0/218)	NA
Congestive heart failure	21.7% (10/46)	16.8% (29/173)	0.515	18.4% (9/49)	8.7% (19/219)	0.067
Cardiomyopathy	6.7% (3/45)	7.5% (13/174)	1	4.1% (2/49)	1.8% (4/218)	0.304
Diabetes	44.7% (21/47)	28.0% (49/175)	0.034	12.0% (6/50)	19.5% (43/220)	0.308
Arthritis	29.5% (13/44)	35.3% (61/173)	0.594	26.0% (13/50)	30.3% (66/218)	0.609
Hypogonadism	2.2% (1/45)	1.8% (3/167)	1	NA	NA	NA
**Family History**						
Family history of HH	8.1% (3/37)	0% (0/157)	0.006	6.7% (3/45)	1.5% (3/199)	0.078
**Medications**						
Over-the-counter arthritis medications	17.0% (8/47)	20.0% (34/170)	0.835	24.0% (12/50)	24.2% (52/215)	1
Oral diabetes medications	27.7% (13/47)	18.6% (32/172)	0.221	7.8% (4/51)	16.0% (35/219)	0.184
Insulin	17.0% (8/47)	14.9% (26/174)	0.820	3.9% (2/51)	13.6% (30/220)	0.056
**Physical Exam**						
Pain on palpation PIP or MCP joints	6.5% (3/46)	3.0% (5/166)	0.375	7.1% (3/42)	3.0% (6/201)	0.190
Skin pigmentation	4.4% (2/45)	1.2% (2/167)	0.199	2.4% (1/42)	2.5% (5/201)	1
Hepatomegaly	2.2% (1/46)	3.6% (6/165)	1	0% (0/41)	2.5% (5/201)	0.592
Ascites	4.3% (2/46)	1.8% (3/167)	0.295	0% (0/42)	2.5% (5/201)	0.591
Testicular atrophy	2.2% (1/45)	0% (0/161)	0.218	NA	NA	NA
**Laboratories**						
AST > 80 u/l	2.5% (1/40)	12.4% (19/153)	0.082	8.5% (4/47)	8.9% (17/191)	1
ALT > 110 u/l	0% (0/35)	7.5% (10/133)	0.124	5.1% (2/39)	8.2% (13/158)	0.740
Transferrin saturation > 50%	100% (9/9)	37.5% (6/16)	0.003	50.0% (4/8)	37.5% (15/40)	0.695
Ferritin > 200 ng/ml (females); > 300 ng/ml (males)	77.8% (14/18)	33.3% (8/24)	0.006	30.8% (4/13)	30.2% (16/53)	1

Clinical features are arranged by genotype. The total number of individuals might vary for different variables as a result of missing or unavailable clinical data. Data are represented as a percentage, and the proportion of affected individuals is in parentheses. Abbreviations are as follows: ALT, alanine aminotransferase; AST, aspartate aminotransferase; HH, hereditary hemochromatosis; MCP, metacarpophalangeal; NA, not available; PIP, proximal interphalangeal.
[a]Diagnosed by notes and ICD-9 code 571.
[b]Diagnosed by notes, ICD-9 codes 571, 794.8, 790.4, and 790.6, and lab criteria (liver enzyme elevation for >6 months).

shown in Table 2, and a comprehensive list of phenotypes is shown in Table S3. The diagnostic rates by genotype and study site are shown in Table S4. The Kaplan-Meier curve (Figure 1) demonstrates the frequency of HH diagnoses by age and sex for p.Cys282Tyr homozygotes and p.[Cys282Tyr];[His63Asp] compound heterozygotes separately.

As expected, for many signs of HH, the penetrance was higher in p.Cys282Tyr homozygotes than in p.[Cys282Tyr];[His63Asp] compound heterozygotes, although some signs

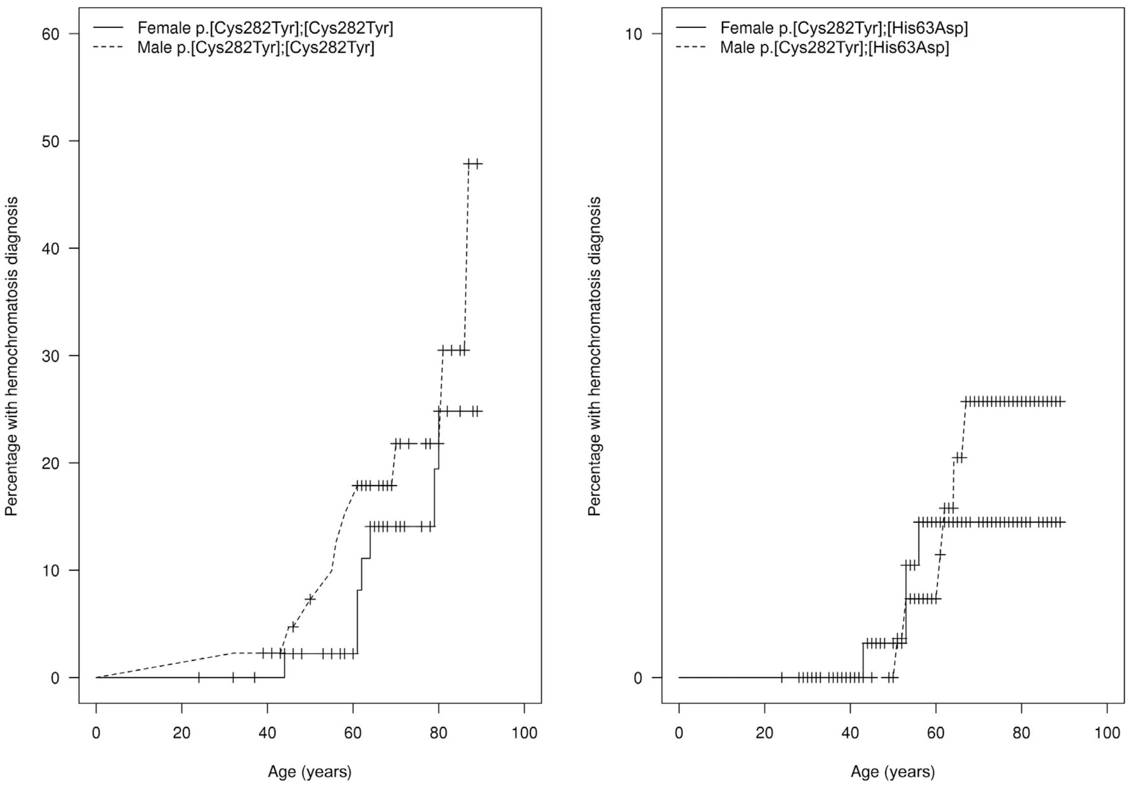

Figure 1. Kaplan-Meier Curves of HH Diagnosis
Frequency of HH diagnosis by age and sex separately for *HFE* p.Cys282Tyr homozygotes and p.[Cys282Tyr];[His63Asp] compound heterozygotes. Each crosshair represents a new HH diagnosis.

of HH did not differ by genotype. Iron studies were significantly different between genotypes in males only, where transferrin saturation above 50% was more common in homozygotes than in compound heterozygotes (100% versus 37.5%; p = 0.003), and serum ferritin higher than 300 ng/ml was more frequent in homozygotes (77.8% versus 33.3%; p = 0.006). No differences in iron studies were found across genotypes in females.

The overall prevalence of liver disease in males was 34.3% in homozygotes and 24.4% in compound heterozygotes (p = 0.279), and in females the prevalence was 29% for both genotypes. The rate of liver biopsy was also significantly different between homozygotes and compound heterozygotes for males (10.9% versus 1.8%; p = 0.013) and females (9.1% versus 2%; p = 0.035). No significant genotype differences were found for other liver phenotypes, which included the presence of any liver disease, liver cirrhosis, other chronic liver diseases, hepatocellular carcinoma, elevated transaminases, or physical findings consistent with liver disease (e.g., hepatomegaly or ascites).

Differences between genotypes were found in the proportion of individuals with a history of phlebotomy in males (19.6% in homozygotes versus 2.9% in compound heterozygotes; p = < 0.001) and females (8% versus 0.5%; p = 0.005), as expected. Males, but not females, had differences between genotypes in the rates of diabetes (44.7% versus 28%; p = 0.03) and family history of HH (8.1% versus 0%; p = 0.006), whereas females had genotype differences in the proportion of hand X-ray for evaluation of arthritis (24.5% versus 11.5%; p = 0.023). No significant differences were found between genotypes for either sex in the rates of congestive heart failure, cardiomyopathy, osteoarthritis, hypogonadism, history of alcohol or tobacco abuse, use of over-the-counter medication for arthritis, diabetes medication (including insulin), proportion of individuals with imaging studies (e.g., abdominal ultrasound or echocardiogram), presence of arthralgia, pain on palpation of proximal interphalangeal or metacarpophalangeal joints, or skin hyperpigmentation.

In one of our study sites (Vanderbilt University), it was noted that among 41 p.Cys282Tyr homozygotes, 7 (17%) were receiving iron, and none of these individuals had a diagnosis of HH. Out of these seven individuals, three had iron labs tested: two had normal studies, and one underwent Roux-en-Y gastric bypass surgery, after which iron was prescribed (iron saturation was initially normal at 37% but then jumped to 71% after 6 months of iron-replacement therapy).

Discussion

In this study, we estimated the diagnostic rate and clinical penetrance of HH in p.Cys282Tyr homozygous and

p.[Cys282Tyr];[His63Asp] compound-heterozygous individuals in the eMERGE Network. We found that among p.Cys282Tyr homozygotes, 24% of male and 14% of female individuals carried a diagnosis of HH. These proportions are higher than the diagnostic rate of 8.5% reported previously in the literature for males and females with this genotype.[13] For compound heterozygotes, 3.4% of males and 2.3% of females were diagnosed with HH. We found that 100% of p.Cys282Tyr homozygotes had a transferrin saturation > 50%, and 78% had ferritin > 300 ng/ml, whereas only 37% and 33% of p.[Cys282Tyr];[His63Asp] heterozygotes met these transferrin and ferritin thresholds, respectively. The biochemical penetrance (i.e., presence of iron overload by transferrin saturation and serum ferritin concentration only) was similar to estimates from previous studies of large prospective cohorts.[8,13,25]

The overall prevalence of liver disease ranged from 24% to 34%, higher than the 10% or lower reported for homozygous males in prior studies.[9,13,26,27] This might be due to our broad definition of liver disease and the inability to separate HH-related liver disease from other causes of liver disease. The prevalence of liver cirrhosis in p.Cys282Tyr homozygous males was 4.5%, consistent with previous reports of 3.4%–5% in this population.[27] The only liver-related phenotype that significantly differed between genotypes was the proportion of individuals who underwent liver biopsy, which was higher for homozygous females and males than for compound heterozygotes. A hypothesis for this difference is that the referral of individuals for liver biopsy happened after a suspicion of hemochromatosis was made on the basis of biochemical (i.e., abnormal iron studies) rater than clinical abnormalities.

Finally, our study revealed that most of what is considered the extreme phenotype of hemochromatosis—which includes liver cirrhosis, hepatocellular carcinoma, and cardiac phenotypes, specifically cardiomyopathies—does not differ across genotypes, which is consistent with the fact that these late manifestations are nowadays uncommon as a consequence of HH and can be prevented by the early detection and treatment of HH with phlebotomy. The exception to this was diabetes, a late HH complication that we found more frequently among homozygous than among compound-heterozygous males. Furthermore, early signs of hemochromatosis (e.g., fatigue, arthritis, and skin hyperpigmentation) did not significantly differ by genotype, possibly because these signs are difficult to define, might not be captured in the electronic health record, and occur commonly in the population at large and thus limit power.

Finding a higher rate of diagnosis of HH in our study has implications for the return of incidental genomic findings. Currently, the return of pathogenic *HFE* variants to individuals who have genomic information available (opportunistic screening) is not yet recommended by the ACMG.[16] Although the adult onset and incomplete penetrance of HH might be inadequate to justify screening of children[14,15] and the ACMG currently does not recommend directed HH testing unless an individual has iron overload or a family history of *HFE*-associated HH, a lower threshold of risk might be considered for opportunistic screening of existing genomic data. New recommendations should address the effectiveness of opportunistic screening and population screening for hemochromatosis under this new evidence, as well as penetrance data from other cohorts unselected for genetic phenotypes and large-scale genetic data linked to medical-record information. Moreover, with the decreasing cost of sequencing, and the additive effectiveness of finding a predisposition not only to hemochromatosis but also to cancer and to other common diseases, the use of genomic tests (i.e., exome or genome sequencing) for newborn or adult screening in the general population might be clinically beneficial. This incorporation of genomic technologies for screening purposes will need to be supported by studies of cost effectiveness and outcomes research.

A second clinical implication results from the possibility of preemptively identifying individuals with a high-risk phenotype and currently receiving medications that might exacerbate a condition or make it clinically evident. A single eMERGE site observed that 17% of individuals with the p.Cys282Tyr homozygous genotype were known to have received oral iron, and one of these individuals developed significant iron overload, possibly as a result of this medication. The finding of common use of iron supplementation in these individuals is not unexpected, given the general frequency of iron supplementation. Had the HH susceptibility of these individuals been known, iron supplementation most likely would have been avoided or more carefully monitored. Although not an original objective of the study, eMERGE sites capable of re-contact are addressing the possible return of HH genotype results to relevant individuals. For other sites, either data are de-identified and re-contact is not possible or affected participants are now deceased. Future prospective studies will need to evaluate clinical outcomes in individuals identified through preemptive diagnosis of hemochromatosis.

From a research perspective, another important implication from our study is that multicenter retrospective cohorts might be useful for determining penetrance estimates of inherited disorders. This idea is supported by the fact that most of our penetrance estimates were consistent with those from studies of prospective hemochromatosis cohorts. Our findings could serve as a proof of concept that linking genotype data to medical-record information might be useful for estimating clinical penetrance of relatively uncommon genotypes associated with common diseases, for example, the estimation of neurologic disease in individuals harboring glucocerebrosidase (*GBA* [MIM: 606463]) variants, which have been found to cause Parkinson disease in Ashkenazi Jews,[28] or the contribution of *BRCA1* (MIM: 113705) and *BRCA2* (MIM: 600185) variants to the development of breast cancer in the general population. Given that several phenotypes could be studied under one cohort, this might also represent a cost-effective method for penetrance estimation.

Our study has several major limitations. First, because this was a retrospective cohort study collecting phenotype and diagnosis data with chart abstractions, the data from the pre-existing clinical records were incomplete. For example, in many cases, imaging data, specific clinical findings, and laboratory values sought for the chart abstraction were not found in the clinical record (see Table S3 for differences in the total number of individuals for each variable). Some clinical sites, such as those with health-maintenance-organization care models, had very complete clinical records, whereas referral centers in general had higher numbers of records missing the data we sought for our purposes, which could have led to underestimation of penetrance. Alternatively, in many participants with a HH diagnosis, it was unclear which criteria were used for diagnosis; it is possible that some of these participants did not manifest HH signs or symptoms but were genotyped specifically for *HFE* variants on the basis of family history of this disorder instead of clinical presentation and were incorrectly diagnosed with HH. Although possible inclusion of participants with a HH family history and predisposing genotype without clinical signs or symptoms might lead to overestimation of the HH diagnostic rate, it does not lead to overestimation of the required clinical care. It can be argued that even if a person lacking signs and symptoms is clinically misdiagnosed with HH because of a family history and genotype, the fact that the test was done and a HH diagnosis was made would support the advantage of opportunistic screening as opposed to clinical screening. It also might be an opportunity for a geneticist or other expert to clarify whether or not the individual is indeed affected by HH.

A second limitation is the lack of a true control group who did not have genotypes at risk for HH. This was due to resource limitations for the chart reviews. Although such a group was not necessary for evaluating diagnostic rates, it would have been useful for determining the baseline rate of the potential HH-related phenotypes, such as liver disease that could not be attributed to HH. Because we expected a lower rate of end-organ damage in the compound heterozygotes, these provided a good comparison to the p.Cys282Tyr homozygotes. The lower clinical and biochemical penetrance of HH in compound heterozygotes than in p.Cys282Tyr homozygous individuals has been documented in large prospective studies, and our estimates were overall consistent with these estimates. For example, in the Pedersen et al. study,[25] a transferrin saturation > 50% was found in 88.9% of p.Cys282Tyr homozygotes and 22.7% of p.[Cys282Tyr];[His63Asp] compound heterozygotes (in comparison to 100% and 37.5%, respectively, in our study). In that same study, the penetrance rate of this phenotype was 14.9% in p.His63Asp homozygous individuals and 5.7% in *HFE* wild-types, confirming the strong effect of genotype on biochemical penetrance. The higher penetrance in p.Cys282Tyr homozygotes than in compound heterozygotes, as well as the even lower penetrance in p.His63Asp homozygotes and *HFE* wild-types, has also been documented for clinical phenotypes, including liver disease, fatigue, and arthritis.[13] The disease penetrance attributable to compound heterozygotes or p.Cys282Tyr homozygotes when compared to individuals with wild-type HFE or single p.Cys282Tyr or p.His63Asp heterozygotes in multicenter cohorts with genotype data linked to medical records will need to be determined by future studies.

A third major limitation was heterogeneity in terms of clinical sites and the subject selection, which was not random when compared to the general population. Our study collected data from seven sites, some of which ascertained people from outpatient clinics (others recruited in hospitals), and there was also heterogeneity in the phenotype selection. To address this limitation, we looked for potential sources of confounding with the HH diagnosis and the pleiotropic effect of iron overload. This concern was mitigated by exclusion of participants ascertained in a liver clinic and the exclusion of liver-biopsy data for those ascertained because of gastric-bypass surgery with concurrent routine liver biopsy.

A fourth limitation is that because of a low number of participants from other races and ethnicities, our analysis was restricted to individuals of European ancestry; thus, our conclusions cannot be generalized to other populations. Future studies will be needed to address the question of clinical penetrance of HH in non-Europeans, who have a considerably lower frequency of HH-causing *HFE* variants.[8]

In summary, we evaluated the diagnostic rate and estimated the penetrance of iron overload and associated organ damage for the two most common HH genotypes in a densely phenotyped cohort selected for reasons unassociated with HH. We found that the rate of HH diagnosis is higher than previously reported and confirmed that the prevalence of iron overload is higher in HFE p.Cys282Tyr homozygotes than in compound heterozygotes. We also found that, compared to large prospective cohorts, unselected multicenter retrospective cohorts might be good models for obtaining penetrance estimates. The use of opportunistic screening for HFE p.Cys282Tyr homozygosity conferring risk of HH in those with existing genomic data should be reconsidered in light of this and future evidence. Furthermore, genetic testing in the general population should be evaluated with studies of outcomes research and cost effectiveness, especially considering the decreasing cost of genomic-technology applications and the additive effectiveness of assessing the risk for multiple adult-onset diseases.

Supplemental Data

Supplemental Data include four tables and can be found with this article online at http://dx.doi.org/10.1016/j.ajhg.2015.08.008.

Acknowledgments

The eMERGE Network was initiated and funded by the National Human Genome Research Institute (NHGRI) through the

following grants: U01HG006828 (Cincinnati Children's Hospital Medical Center and Boston Children's Hospital), U01HG006830 (Children's Hospital of Philadelphia), U01HG006389 (Essentia Institute of Rural Health, Marshfield Clinic Research Foundation, and Pennsylvania State University), U01HG006382 (Geisinger Clinic), U01HG006375 (Group Health Cooperative and the University of Washington), U01HG006379 (Mayo Clinic), U01HG006380 (Icahn School of Medicine at Mount Sinai), U01HG006388 (Northwestern University), U01HG006378 (Vanderbilt University Medical Center), and U01HG006385 (Vanderbilt University Medical Center serving as the coordinating center). G.P.J. was supported by grant 5T32GM007454 from the National Institute of General Medical Sciences and grants U01HG0006507 and U01HG007307 from the NHGRI and National Cancer Institute. C.J.G. was supported by grant K12 HS021686 from the Patient-Centered Outcomes Research Career Development Program of the Agency for Healthcare Research and Quality.

Received: April 26, 2015
Accepted: August 17, 2015
Published: September 10, 2015

Web Resources

The URLs for data presented herein are as follows:

OMIM, https://www.omim.org/
RefSeq, http://www.ncbi.nlm.nih.gov/refseq/
Research Electronic Data Capture, http://project-redcap.org/
R project for statistical computing, http://www.r-project.org/

References

1. Bacon, B.R., Adams, P.C., Kowdley, K.V., Powell, L.W., and Tavill, A.S.; American Association for the Study of Liver Diseases (2011). Diagnosis and management of hemochromatosis: 2011 practice guideline by the American Association for the Study of Liver Diseases. Hepatology *54*, 328–343.
2. Niederau, C., Fischer, R., Pürschel, A., Stremmel, W., Häussinger, D., and Strohmeyer, G. (1996). Long-term survival in patients with hereditary hemochromatosis. Gastroenterology *110*, 1107–1119.
3. Feder, J.N., Gnirke, A., Thomas, W., Tsuchihashi, Z., Ruddy, D.A., Basava, A., Dormishian, F., Domingo, R., Jr., Ellis, M.C., Fullan, A., et al. (1996). A novel MHC class I-like gene is mutated in patients with hereditary haemochromatosis. Nat. Genet. *13*, 399–408.
4. Pietrangelo, A. (2010). Hereditary hemochromatosis: pathogenesis, diagnosis, and treatment. Gastroenterology *139*, 393–408, 408.e1–408.e2.
5. van Bokhoven, M.A., van Deursen, C.T., and Swinkels, D.W. (2011). Diagnosis and management of hereditary haemochromatosis. BMJ *342*, c7251.
6. Bardou-Jacquet, E., Ben Ali, Z., Beaumont-Epinette, M.P., Loreal, O., Jouanolle, A.M., and Brissot, P. (2014). Non-HFE hemochromatosis: pathophysiological and diagnostic aspects. Clin. Res. Hepatol. Gastroenterol. *38*, 143–154.
7. Waalen, J., Felitti, V., Gelbart, T., Ho, N.J., and Beutler, E. (2002). Penetrance of hemochromatosis. Blood Cells Mol. Dis. *29*, 418–432.
8. Adams, P.C., Reboussin, D.M., Barton, J.C., McLaren, C.E., Eckfeldt, J.H., McLaren, G.D., Dawkins, F.W., Acton, R.T., Harris, E.L., Gordeuk, V.R., et al.; Hemochromatosis and Iron Overload Screening (HEIRS) Study Research Investigators (2005). Hemochromatosis and iron-overload screening in a racially diverse population. N. Engl. J. Med. *352*, 1769–1778.
9. Beutler, E., Felitti, V.J., Koziol, J.A., Ho, N.J., and Gelbart, T. (2002). Penetrance of 845G–> A (C282Y) HFE hereditary haemochromatosis mutation in the USA. Lancet *359*, 211–218.
10. Olynyk, J.K., Cullen, D.J., Aquilia, S., Rossi, E., Summerville, L., and Powell, L.W. (1999). A population-based study of the clinical expression of the hemochromatosis gene. N. Engl. J. Med. *341*, 718–724.
11. Andersen, R.V., Tybjaerg-Hansen, A., Appleyard, M., Birgens, H., and Nordestgaard, B.G. (2004). Hemochromatosis mutations in the general population: iron overload progression rate. Blood *103*, 2914–2919.
12. Delatycki, M.B., Allen, K.J., Nisselle, A.E., Collins, V., Metcalfe, S., du Sart, D., Halliday, J., Aitken, M.A., Macciocca, I., Hill, V., et al. (2005). Use of community genetic screening to prevent HFE-associated hereditary haemochromatosis. Lancet *366*, 314–316.
13. Allen, K.J., Gurrin, L.C., Constantine, C.C., Osborne, N.J., Delatycki, M.B., Nicoll, A.J., McLaren, C.E., Bahlo, M., Nisselle, A.E., Vulpe, C.D., et al. (2008). Iron-overload-related disease in HFE hereditary hemochromatosis. N. Engl. J. Med. *358*, 221–230.
14. Whitlock, E.P., Garlitz, B.A., Harris, E.L., Beil, T.L., and Smith, P.R. (2006). Screening for hereditary hemochromatosis: a systematic review for the U.S. Preventive Services Task Force. Ann. Intern. Med. *145*, 209–223.
15. Cadet, E., Capron, D., Gallet, M., Omanga-Léké, M.L., Boutignon, H., Julier, C., Robson, K.J., and Rochette, J. (2005). Reverse cascade screening of newborns for hereditary haemochromatosis: a model for other late onset diseases? J. Med. Genet. *42*, 390–395.
16. Green, R.C., Berg, J.S., Grody, W.W., Kalia, S.S., Korf, B.R., Martin, C.L., McGuire, A.L., Nussbaum, R.L., O'Daniel, J.M., Ormond, K.E., et al.; American College of Medical Genetics and Genomics (2013). ACMG recommendations for reporting of incidental findings in clinical exome and genome sequencing. Genet. Med. *15*, 565–574.
17. Amendola, L.M., Dorschner, M.O., Robertson, P.D., Salama, J.S., Hart, R., Shirts, B.H., Murray, M.L., Tokita, M.J., Gallego, C.J., Kim, D.S., et al. (2015). Actionable exomic incidental findings in 6503 participants: challenges of variant classification. Genome Res. *25*, 305–315.
18. McCarty, C.A., Chisholm, R.L., Chute, C.G., Kullo, I.J., Jarvik, G.P., Larson, E.B., Li, R., Masys, D.R., Ritchie, M.D., Roden, D.M., et al.; eMERGE Team (2011). The eMERGE Network: a consortium of biorepositories linked to electronic medical records data for conducting genomic studies. BMC Med. Genomics *4*, 13.
19. Gottesman, O., Kuivaniemi, H., Tromp, G., Faucett, W.A., Li, R., Manolio, T.A., Sanderson, S.C., Kannry, J., Zinberg, R., Basford, M.A., et al.; eMERGE Network (2013). The Electronic Medical Records and Genomics (eMERGE) Network: past, present, and future. Genet. Med. *15*, 761–771.
20. Roden, D.M., Pulley, J.M., Basford, M.A., Bernard, G.R., Clayton, E.W., Balser, J.R., and Masys, D.R. (2008). Development of a large-scale de-identified DNA biobank to enable personalized medicine. Clin. Pharmacol. Ther. *84*, 362–369.
21. Turner, S., Armstrong, L.L., Bradford, Y., Carlson, C.S., Crawford, D.C., Crenshaw, A.T., de Andrade, M., Doheny, K.F., Haines, J.L., Hayes, G., et al. (2011). Quality control

procedures for genome-wide association studies. Curr. Protoc. Hum. Genet. *Chapter 1*, Unit 1.19.

22. Verma, S.S., de Andrade, M., Tromp, G., Kuivaniemi, H., Pugh, E., Namjou-Khales, B., Mukherjee, S., Jarvik, G.P., Kottyan, L.C., Burt, A., et al. (2014). Imputation and quality control steps for combining multiple genome-wide datasets. Front. Genet. *5*, 370.
23. Banks, N.J. (1998). Designing medical record abstraction forms. Int. J. Qual. Health Care *10*, 163–167.
24. Harris, P.A., Taylor, R., Thielke, R., Payne, J., Gonzalez, N., and Conde, J.G. (2009). Research electronic data capture (REDCap)–a metadata-driven methodology and workflow process for providing translational research informatics support. J. Biomed. Inform. *42*, 377–381.
25. Pedersen, P., and Milman, N. (2009). Genetic screening for HFE hemochromatosis in 6,020 Danish men: penetrance of C282Y, H63D, and S65C variants. Ann. Hematol. *88*, 775–784.
26. Rossi, E., and Jeffrey, G.P. (2004). Clinical penetrance of C282Y homozygous HFE haemochromatosis. Clin. Biochem. Rev. *25*, 183–190.
27. Asberg, A., Hveem, K., Kannelønning, K., and Irgens, W.O. (2007). Penetrance of the C28Y/C282Y genotype of the HFE gene. Scand. J. Gastroenterol. *42*, 1073–1077.
28. Aharon-Peretz, J., Rosenbaum, H., and Gershoni-Baruch, R. (2004). Mutations in the glucocerebrosidase gene and Parkinson's disease in Ashkenazi Jews. N. Engl. J. Med. *351*, 1972–1977.

The American Journal of Human Genetics

Supplemental Data

Penetrance of Hemochromatosis in *HFE* Genotypes Resulting in p.Cys282Tyr and p.[Cys282Tyr];[His63Asp] in the eMERGE Network

Carlos J. Gallego, Amber Burt, Agnes S. Sundaresan, Zi Ye, Christopher Shaw, David R. Crosslin, Paul K. Crane, S. Malia Fullerton, Kris Hansen, David Carrell, Helena Kuivaniemi, Kimberly Derr, Mariza de Andrade, Catherine A. McCarty, Terrie E. Kitchner, Brittany K. Ragon, Sarah C. Stallings, Gabriella Papa, Joseph Bochenek, Maureen E. Smith, Sharon A. Aufox, Jennifer A. Pacheco, Vaibhav Patel, Elisha M. Friesema, Angelika Ludtke Erwin, Omri Gottesman, Glenn S. Gerhard, Marylyn Ritchie, Arno G. Motulsky, Iftikhar J. Kullo, Eric B. Larson, Gerard Tromp, Murray H. Brilliant, Erwin Bottinger, Joshua C. Denny, Dan M. Roden, Marc S. Williams, and Gail P. Jarvik

Table S1. Genotyped versus imputed variants by site. Number of samples that were genotyped or imputed for *HFE* variants p.Cys282Tyr and p.His63Asp by site. [a] Only p.Cys282Tyr was genotyped, p.His63Asp was imputed. ; [b] Only p.His63Asp was genotyped, p.Cys282Tyr was imputed.; [c] Both p.Cys282Tyr and p.His63Asp were imputed.

GENOTYPED SNP					
SITE	BOTH	p.Cys282Tyr[a]	p.His63Asp[b]	NEITHER[c]	TOTAL
Geisinger	974	0	3111	0	4085
Group Health/UW	0	3130	398	0	3528
Marshfield	0	4193	0	0	4193
Mayo	14	6862	0	0	6876
Mt. Sinai	0	2775	3515	0	6290
Northwestern	605	1302	0	2951	4858
Vanderbilt	2082	4004	0	3461	9547
Total	3675	22266	7024	6412	39377

Table S2. Examples of data obtained in chart abstraction, by category. The list is in the order of information usually present in medical records.

DATA CATEGORY	EXAMPLES
Demographic	Age at last observation, vital status, sex, race, ethnicity
Medical history	Hemochromatosis, iron-overload, liver cirrhosis, chronic liver diseases, secondary causes of hemochromatosis, congestive heart failure, cardiomyopathy, diabetes, arthritis, hypogonadism
Laboratories	Transferrin saturation, serum ferritin, serum iron, total iron binding capacity, liver transaminases, prothrombin time, partial thromboplastin time, follicle-stimulating hormone, luteinizing hormone, testosterone
Imaging	Hand x-ray, echocardiogram, cardiac magnetic resonance imaging, abdominal ultrasound, abdominal computed tomography, electrocardiogram
Family history	Relatives affected with hemochromatosis, number and degree of relationship
Past social history	Alcoholism, tobacco abuse, intravenous drug use
Medications	Tylenol, other arthritis medications, iron supplements, diabetes medications
Physical exam	Hepatomegaly, ascites, signs of congestive heart failure, arthritis, skin pigmentation (bronze skin)

Table S3. List of covariates in the analysis, stratified by genotype and sex. The list is in the order of information usually present in medical records: medical, family, social history, medications, review of systems, imaging studies, laboratories, and physical findings. Italicized text corresponds to liver biopsy characteristics. Unless otherwise specified, data are represented as percentage, with the proportion of affected patients in parentheses. Abbreviations: NA, not available; AST, aspartate aminotransferase; ALT, alanine aminotransferase; HH, hereditary hemochromatosis; PIP/MCP, proximal interphalangeal/metacarpophalangeal. Footnotes: [a] Diagnosed by notes, ICD-9 code 275.0 or 275.09 or lab criteria: transferrin saturation (> 45%) and serum ferritin (>200 ng/mL in men and >150 ng/mL in women), [b] Diagnosed by notes, ICD-9 code 571, [c] Diagnosed by notes, ICD-9 codes 571, 794.8, 790.4, 790.6, lab criteria: liver enzyme elevation for >6 months, [d] Diagnosed by notes, ICD-9 codes 280.9 or 275.0, lab criteria: ferritin <40 ng/mL and anemia or ICD-9 codes 281 or 285.

	p.Cys282Tyr/p.Cys282Tyr (N=98)			p.Cys282Tyr/p.His63Asp (N=397)		
VARIABLE	N	MALE (N=47)	FEMALE (N=51)	N	MALE (N=175)	FEMALE (N=222)
Body Mass Index	83	29.0±5.4	32.3±23.5	359	29.0±4.9	29.4±8.2
Liver Biopsy (any liver biopsy)	90	10.9% (5/46)	9.1% (4/44)	371	1.8% (3/166)	16.1% (33/205)
Liver Biopsy (not incidental to gastric bypass)	90	10.9% (5/46)	9.1% (4/44)	371	1.8% (3/166)	2.0% (4/205)
Cirrhosis in Liver Biopsy Results	6	0.0% (0/3)	33.3% (1/3)	38	0.0% (0/4)	5.9% (2/34)
Normal Liver Biopsy Results	5	0% (0/3)	50% (1/2)	35	0.0% (0/3)	18.8% (6/32)
Iron Deposition in Liver Biopsy Results	7	33.3% (1/3)	50.0% (2/4)	35	66.7% (2/3)	28.1% (9/32)
Iron Overload [a]	88	30.4% (14/46)	9.5% (4/42)	375	3.5% (6/170)	2.9% (6/205)

Liver Cirrhosis [b]	84	4.5% (2/44)	2.5% (1/40)	369	4.8% (8/166)	4.9% (10/203)
Other Chronic Liver Disease Causes [c]	84	7% (3/43)	0% (0/41)	369	6.7% (11/164)	7.8% (16/205)
Iron Deficiency [d]	86	15.6% (7/45)	4.9% (2/41)	371	7.1% (12/168)	10.8% (22/203)
Phlebotomy	96	19.6% (9/46)	8.0% (4/50)	391	2.9% (5/172)	0.5% (1/219)
Congestive Heart Failure	95	21.7% (10/46)	18.4% (9/49)	392	16.8% (29/173)	8.7% (19/219)
Cardiomyopathy	94	6.7% (3/45)	4.1% (2/49)	392	7.5% (13/174)	1.8% (4/218)
Coronary Artery Disease	92	29.5% (13/44)	12.5% (6/48)	388	28.1% (48/171)	11.1% (24/217)
Diabetes	97	44.7% (21/47)	12.0% (6/50)	395	28.0% (49/175)	19.5% (43/220)
Arthritis	94	29.5% (13/44)	26.0% (13/50)	391	35.3% (61/173)	30.3% (66/218)
Hepatocellular Carcinoma	96	0% (0/46)	0% (0/50)	387	0% (0/169)	0% (0/218)
Family History of HH	82	8.1% (3/37)	6.7% (3/45)	356	0.0% (0/157)	1.5% (3/199)
Alcohol Dependence	94	13.3% (6/45)	2.0% (1/49)	381	10.6% (18/170)	7.1% (15/211)
Tobacco Use	94	30.4% (14/46)	16.7% (8/48)	391	31.6% (55/174)	23.0% (50/217)
Intravenous Drug Use	84	0% (0/40)	0% (0/44)	346	0.7% (1/150)	1.0% (2/196)
Over the counter Arthritis Medication	97	17% (8/47)	24% (12/50)	385	20.0% (34/170)	24.2% (52/215)
Acetaminophen	97	26.1% (12/46)	45.1% (23/51)	388	32.2% (55/171)	39.2% (85/217)
Prescription Arthritis Medication	95	25.0% (11/44)	17.6% (9/51)	384	30.4% (51/168)	26.9% (58/216)
Erectile Dysfunction Medication	47	19.1% (9/47)	NA	172	21.5% (37/172)	NA
Insulin	98	17.0% (8/47)	3.9% (2/51)	394	14.9% (26/174)	13.6% (30/220)
Oral Diabetes Therapy	98	27.7% (13/47)	7.8% (4/51)	391	18.6% (32/172)	16.0% (35/219)
Chronic Weakness/Lethargy	89	14.9% (7/47)	16.7% (7/42)	371	5.3% (9/169)	9.9% (20/202)
Arthralgia	90	25.5% (12/47)	23.3% (10/43)	373	24.0% (41/171)	21.8% (44/202)
Impotence	45	26.7% (12/45)	NA	158	16.5% (26/158)	NA
Abdominal Pain	87	17.8% (8/45)	7.1% (3/42)	365	9.6% (16/166)	18.1% (36/199)
Hepatomegaly	87	2.2% (1/46)	0.0% (0/41)	366	3.6% (6/165)	2.5% (5/201)
Ascites	88	4.3% (2/46)	0.0% (0/42)	368	1.8% (3/167)	2.5% (5/201)
Pain on palpation PIP/MCP joints	88	6.5% (3/46)	7.1% (3/42)	367	3% (5/166)	3% (6/201)

Skin Pigmentation	87	4.4% (2/45)	2.4% (1/42)	368	1.2% (2/167)	2.5% (5/201)
Hypogonadism	45	2.2% (1/45)	NA	167	1.8% (3/167)	NA
Erectile Dysfunction	42	23.8% (10/42)	NA	166	27.1% (45/166)	NA
Testicular Atrophy	45	2.2% (1/45)	NA	161	0% (0/161)	NA
Gynecomastia	43	0% (0/43)	NA	161	1.2% (2/161)	NA
Hand X-Ray	95	10.9% (5/46)	24.5% (12/49)	390	18.5% (32/173)	11.5% (25/217)
Echocardiogram	95	52.2% (24/46)	44.9% (22/49)	387	58.5% (100/171)	42.1% (91/216)
Cardiac Magnetic Resonance Imaging	96	2.2% (1/46)	0.0% (0/50)	390	0.6% (1/173)	0.9% (2/217)
Abdominal Ultrasound	94	25% (11/44)	26% (13/50)	392	24.7% (43/174)	34.4% (75/218)
Cirrhosis on Abdominal Ultrasound	25	0% (0/11)	0% (0/14)	118	4.7% (2/43)	2.7% (2/75)
Computed Tomography of Abdomen	96	41.3% (19/46)	26.0% (13/50)	389	46.5% (80/172)	37.3% (81/217)
Electrocardiogram	94	79.1% (34/43)	78.4% (40/51)	384	90.5% (153/169)	83.3% (179/215)
Highest AST (u/L)	87	41.5±20.1	50.7±82.6	344	87.4±354.1	51.3±94.1
AST > 80 u/L	87	2.5% (1/40)	8.5% (4/47)	344	12.4% (19/153)	8.9% (17/191)
Highest ALT (u/L)	74	43.3±23.7	68.0±164.2	291	66.7±197.4	57.8±115.6
ALT > 110 u/L	74	0.0% (0/35)	5.1% (2/39)	291	7.5% (10/133)	8.2% (13/158)
Rales on lung exam	87	13.3% (6/45)	14.3% (6/42)	371	6.0% (10/168)	6.4% (13/203)
Lower Extremity Edema	88	17.4% (8/46)	19.0% (8/42)	370	18.5% (31/168)	15.3% (31/202)
Transferrin Sat > 45%	17	100.0% (9/9)	62.5% (5/8)	56	37.5% (6/16)	40.0% (16/40)
Transferrin Sat > 50%	17	100% (9/9)	50% (4/8)	56	37.5% (6/16)	37.5% (15/40)
Highest Transferrin Saturation (%)	17	92.8±4.5	54.9±30.3	56	53.7±52.0	43.2±23.8
Highest Serum Iron Concentration μg/dL	33	186.8±57.8	144.6±58.3	104	127.5±63.2	127.8±60.5
Highest Total Iron-Binding Capacity (ng/mL)	33	269.1±55.5	269.4±42.9	93	288.4±72.8	317.3±61.8
Ferritin > 150 ng/mL	31	88.9% (16/18)	46.2% (6/13)	77	50.0% (12/24)	41.5% (22/53)
Ferritin > 200 ng/mL	31	88.9% (16/18)	30.8% (4/13)	77	45.8% (11/24)	30.2% (16/53)

Ferritin > 300 ng/mL	31	77.8% (14/18)	23.1% (3/13)	77	33.3% (8/24)	18.9% (10/53)
Ferritin > 1000 ng/mL	31	27.8% (5/18)	0.0% (0/13)	77	8.3% (2/24)	1.9% (1/53)
Highest Ferritin Level (ng/mL)	31	762.4±652.6	225.3±218.6	77	316.6±394.0	451.3±1891.9
Highest INR	59	2.8±2.3	2.0±1.2	193	2.2±1.7	2.2±1.8

Table S4. Penetrance of hereditary hemochromatosis by *HFE* genotype and eMERGE site. Data are represented as percentage, with the proportion of affected patients in parentheses.

	MALE		FEMALE	
SITE	p.Cys282Tyr/ p.Cys282Tyr	p.Cys282Tyr/ p.His63Asp	p.Cys282Tyr/ p.Cys282Tyr	p.Cys282Tyr/ p.His63Asp
Geisinger	0% (0/9)	4.4% (1/23)	0% (0/2)	2.6% (1/38)
Group Health/UW	33.3% (1/3)	0% (0/21)	12.5% (1/8)	0% (0/25)
Marshfield	42.9% (3/7)	3.6% (1/28)	25.0% (2/8)	0% (0/23)
Mayo	35.7% (5/14)	3.3% (2/61)	20.0% (3/15)	1.9% (1/53)
Mt Sinai	0% (0/0)	0% (0/0)	0% (0/0)	0% (0/0)
Northwestern	33.3% (1/3)	14.3% (1/7)	14.3% (1/7)	2.6% (1/39)
Vanderbilt	11.1% (1/9)	2.9% (1/34)	0% (0/10)	5.0% (2/40)
Total	24.4% (11/45)	3.5% (6/174)	14.0% (7/50)	2.3% (5/218)
